# Differential age-related changes in N170 responses to upright faces, inverted faces, and eyes in Japanese children

**DOI:** 10.3389/fnhum.2015.00263

**Published:** 2015-06-02

**Authors:** Kensaku Miki, Yukiko Honda, Yasuyuki Takeshima, Shoko Watanabe, Ryusuke Kakigi

**Affiliations:** ^1^Department of Integrative Physiology, National Institute for Physiological SciencesOkazaki, Japan; ^2^Department of Physiological Sciences, School of Life Science, SOKENDAI (The Graduate University for Advanced Studies)Hayama, Japan

**Keywords:** N170, face, development, EEG, eyes, inversion

## Abstract

The main objectives of this study were to investigate the development of face perception in Japanese children, focusing on the changes in face processing strategies (holistic and/or configural vs. feature-based) that occur during childhood. To achieve this, we analyzed the face-related N170 component, evoked by upright face, inverted face, and eyes stimuli in 82 Japanese children aged between 8- and 13-years-old. During the experiment, the children were asked to perform a target detection task in which they were told to press a button when they saw images of faces or kettles with mustaches, glasses, and fake noses; i.e., an implicit face perception task. The N170 signals observed after the presentation of the upright face stimuli were longer in duration and/or had at least two peaks in the 8–11-year-old children, whereas those seen in the 12–13-year-old children were sharp and only had a single peak. N170 latency was significantly longer after the presentation of the eyes stimuli than after the presentation of the upright face stimuli in the 10- and 12-year-old children. In addition, significant differences in N170 latency were observed among all three stimulus types in the 13-year-old children. N170 amplitude was significantly greater after the presentation of the eyes stimuli than after the presentation of the upright face stimuli in the 8–10- and 12-year-old children. The results of the present study indicate that the upright face stimuli were processed using holistic and/or configural processing by the 13-year-old children.

## Introduction

The face contains a lot of information that is relevant to our daily lives, such as information about age, sex, and familiarity, and plays an important role in social communication. Accordingly, the face has been extensively examined in many previous psychological studies. For example, Bruce and Young (1986) described seven codes that can be distinguished during face processing, which they named pictorial, structural, identity-specific semantic, visually-derived semantic, name, expression, and facial speech codes; i.e., the face recognition model. In addition, three types of information are known to be important for face perception (Lee et al., 2013; Liu et al., 2013). The first type is isolated featural information, such as the size of the eyes. The second is configural information, which refers to the spatial relationships between facial features, and the third is holistic information referring to the facial gestalt, which represents the fusion of featural and configural information into an unbroken whole (Tanaka and Farah, 1993).

It has been reported that faces are processed using holistic and/or configural strategies rather than feature-based strategies, which are generally used for object perception (Maurer et al., 2002). In addition, there is phenomenon unique to humans and non-human primates. Psychological studies have reported that face recognition was more difficult when inverted faces were presented rather than upright faces and named this phenomenon the face inversion effect. These findings suggest that face inversion might disrupt the holistic and/or configural processing of facial information (Tanaka and Farah, 1993; Mondloch et al., 2002).

EEG demonstrated that a negative component is evoked at approximately 170 ms during object perception, and this component was termed N170 (Bentin et al., 1996; George et al., 1996). N170 was shown to be larger during the viewing of faces than during the observation of other objects, such as cars or chairs (Rossion and Jacques, 2008), and was found to exhibit longer latency and a greater amplitude when eyes were being examined than during the viewing of upright faces (Watanabe et al., 1999). Therefore, N170 has been proposed to reflect holistic and/or configural processing during face perception. In previous studies, N170 was found to display longer latency and a greater amplitude during the observation of inverted faces than during the viewing of upright faces (Watanabe et al., 2003; Honda et al., 2007); therefore, N170 appears to be modulated by facial inversion, possibly because facial inversion disrupts holistic and/or configural processing and forces featural processing to be employed (Maurer et al., 2002; Rossion and Gauthier, 2002). In addition, recent studies based on event-related potential (ERP) and eye-tracking data have shown the importance of the eyes for face perception processing (Meaux et al., 2014; Nemrodov et al., 2014). For example, it was reported that the amplitude of N170 was greater when the subject fixated on the eyes than when they examined other locations (the forehead, nasion, nose, or mouth) (Nemrodov et al., 2014).

Some researchers have studied the development of face perception using neuroimaging methods (e.g., Lee et al., 2013). Many EEG-based studies have detected changes in N170 with age (Taylor et al., 1999, 2004; de Haan et al., 2002; Itier and Taylor, 2004a,b). In an infant study (de Haan et al., 2002), a putative “infant N170” signal was found to be sensitive to the species of animal to which the presented face belonged; however, the orientation of the face did not influence processing until a later stage, which differed from the findings obtained in adults. The inversion effect does not seem to affect the latency of N170 until 8–11 years of age and does not appear to affect the amplitude of N170 until 13–14 years of age (Taylor et al., 2004). Batty and Taylor (2006) also showed that the sensitivity of N170 to emotions develops late; i.e., at 14- to 15-years-old. A recent study that examined EEG and eye-tracking data detected a correlation between initial fixation on the eyes and N170 and suggested that this correlation was partially driven by common developmental dynamics (Meaux et al., 2014). In an fMRI study, adolescents exhibited face-related activity in the fusiform face area (FFA), occipital face area (OFA), and superior temporal sulcus (STS), which were similar to the regions that were activated in the adult group, whereas none of these face-related regions were activated in the children (Scherf et al., 2007).

Cultural differences in face processing mechanisms are known to exist. Blais et al. (2008) described cultural differences in eye movements between Western Caucasians and East Asians during the learning, recognition, and categorization of faces. In addition, a recent fMRI study found differences between the face processing mechanisms of Western individuals and East Asians; i.e., they detected an analytical style of face processing in the Western subjects and a holistic processing style in the East Asians (Goh et al., 2010).

In this study, we mainly investigated the development of face perception in children, focusing on the changes in face processing strategies (holistic and/or configural vs. feature-based) that occur during childhood. We also compared our results for Japanese children with the findings for Western children reported in previous studies. Based on the findings of previous developmental studies that examined EEG and eye-tracking data, we mainly focused on the N170 component as a developmental marker of face perception processing in this study (Taylor et al., 1999, 2004; Itier and Taylor, 2004a,b; Meaux et al., 2014). On the other hand, P100 is considered to reflect basic and early processing, e.g., responses to changes in luminance, visual field, and visual size (see Meaux et al., 2014). Some studies have detected face-related effects on P100 (Batty and Taylor, 2003; Itier and Taylor, 2004a; Taylor et al., 2004). However, whilst age-related changes in N170 are considered to reflect the development of face perception processing it has been suggested that age-related changes in P100 reflect the general development of sensory and/or cognitive function, e.g., the development of visual acuity or visual attention, etc. (Pastò and Burack, 1997; Skoczenski and Norcia, 2002; Want et al., 2003; Betts et al., 2006; Crookes and McKone, 2009). Therefore, we also analyzed the changes in the P100 component that occur during childhood and compared them with the changes in N170 during the same period. Eighty-two subjects were analyzed in this study after being classified into 6 age groups (into 8, 9, 10, 11, 12, and 13-year-olds), which differed from the method used in previous EEG studies, in which the subjects were divided into two-year age groups (into 4–5, 6–7, 8–9, 10–11, 12–13, and 14–15-year-olds) (Taylor et al., 1999, 2004; Itier and Taylor, 2004a,b). This was the first study to investigate the development of face perception in a large number of Japanese children.

## Methods and Materials

### Subjects

Ninety-one normal right-handed volunteers with normal or corrected visual acuity participated in this study. However, two 8-year-olds, two 9-year-olds, two 12-year-olds, and three 13-year-olds were excluded from the ERP analysis because of artifactual EEG contamination. Therefore, the ERP data of 82 subjects were analyzed.

The 82 subjects were divided into 6 age groups; i.e., into 8-year-olds (*n* = 11, 3 males, mean age: 8.6 ± 0.24-years-old), 9-year-olds (*n* = 17, 7 males, mean age: 9.4 ± 0.22-years-old), 10-year-olds (*n* = 15, 10 males, mean age: 10.2 ± 0.15-years-old), 11-year-olds (*n* = 12, 4 males, mean age: 11.1 ± 0.27-years-old), 12-year-olds (*n* = 10, 7 males, mean age: 12.5 ± 0.20-years-old), and 13-year-olds (*n* = 17, 8 males, mean age: 13.4 ± 0.30-years-old). The subjects were recruited from a primary school and a junior high school in Okazaki city, Aichi Prefecture, Japan. All of the subjects were in age-appropriate levels at school, and none of them had learning or attention problems.

All of the subjects and their parents gave their informed consent to participate in the experiment, which was approved by the ethics committee of the National Institute for Physiological Sciences. All of the experiments were conducted according to the Declaration of Helsinki. Each of the subjects was given a reward at the end of the experiment.

### Visual Stimuli

**Figure 1:**
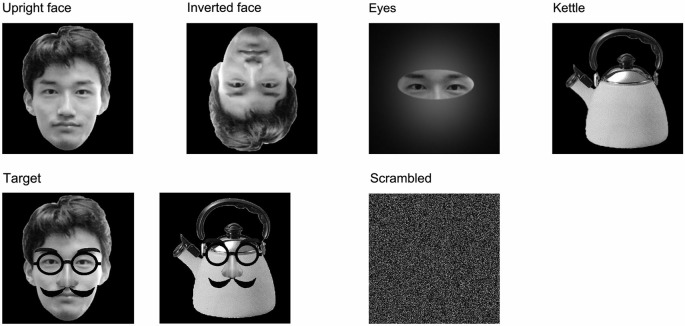
**Examples of the stimuli and other images used in this study**. (1) Upright face: images of a neutral face; (2) Inverted face: inverted versions of the upright face stimuli; (3) Eyes: images showings eyes alone without facial contours or other features; (4) Kettle: images of a kettle with a lid and handle; (5) Target: images of upright faces or kettles with mustaches, glasses, and fake noses; and (6) Scrambled: this image was made by replacing the 160,000 pixels in the stimulus images with faces.

We presented the following five types of stimuli to the children (Figure 1):

(1) Upright face stimuli: images of a neutral face.
(2) Inverted face stimuli: inverted versions of the upright face stimuli.
(3) Eyes stimuli: images showings eyes alone without facial contours or other features.
(4) Kettle stimuli: images of a kettle with a lid and handle.
(5) Target stimuli: images of upright faces or kettles with mustaches, glasses, and fake noses. We considered that the target stimuli would be more interesting for children, especially small children, than non-facial stimuli, such as cars, chairs, flowers, butterflies, or animals, and thus, the presentation of the target stimuli might have helped to minimize habituation and drowsiness. The subjects were asked to push a button as quickly as possible when the target stimuli were presented.

We presented the kettle and target stimuli to ensure that the experimental task acted as an implicit face perception task, and the luminance and contrast of the kettle stimuli differed from those of the upright face, inverted face, and eyes stimuli. Therefore, we analyzed the results obtained under conditions (1), (2), and (3).

The upright face, inverted face, eyes, and kettle stimuli each consisted of 50 different images, and thirty different images were used for the target stimuli. The upright and inverted face stimuli did not have mustaches or glasses. All of the images were gray-scaled and unfamiliar to the subjects. The stimuli were shown for a relatively short period (250 ms) in order to minimize the influence of artifacts, and the inter-stimulus interval lasted for 1000–1200 ms. The stimuli were presented in random order, and a scrambled image, which was made by replacing the 160,000 pixels in the stimulus images with faces, was presented throughout the inter-stimulus interval to minimize the changes in luminance and contrast among the upright face, inverted face, and eyes stimuli (Figure 1). In addition, we asked the subjects to blink during the presentation of the scrambled image. Therefore, each trial took 1250–1450 ms.

The stimuli and scrambled images measured 9.6 degrees × 9.6 degrees and were presented using a personal computer (DELL Dimension XPS T750r) and a monitor (Sony GDM-F520). A red light that measured 0.2 degrees in diameter and was located 140 cm from the subject’s eyes was presented as a fixation point throughout the experiment. The fixation point, stimuli, and scrambled image were presented in the center of the monitor. The subjects were seated on a chair and were instructed to concentrate on the fixation point during the experiment.

To minimize habituation and drowsiness, each subject took part in more than 10 short-term recording sessions. Each recording session included 19–21 trials of the upright face, inverted face, eyes, and kettle stimuli, and 3–5 trials of the target stimuli. In total, the experiment took less than 30 min. Each session was delivered in a pseudorandom order among the subjects.

### Event-Related Potential (ERP) Recording and Data Analysis

ERP were recorded by averaging EEG using a Neuropack MEB 2200 system (Nihon Kohden, Tokyo, Japan) with non-polarizable Ag/AgCl electrodes. EEG electrodes were placed at Fz, Cz, T3, T4, C3, C4, Pz, P3, P4, T5, T6, O1, and O2 based on the International 10–20 System, and additional electrodes were placed at T5’ (2 cm below T5) and T6’ (2 cm below T6) (Taylor et al., 1999; Watanabe et al., 1999). The reference electrode was placed on the tip of the nose, and the ground electrode was placed on the forehead. An electrooculogram (EOG) was also recorded using an electrode located above the right eye and the reference electrode in order to assess the subjects’ blinking and eye movements. The impedance of all electrodes was kept at less than 5 kΩ. EEG and EOG were recorded simultaneously with a band-pass of 0.1–50 Hz, and digitized at a rate of 1000 Hz. By using only a 0.1–50 Hz band-pass filter, the noise above 50 Hz was not completely removed. Thus, we used a 60 Hz AC filter, to remove such noise. The time window for the recording ran from 100 ms before to 400 ms after stimulus onset in order to minimize the influence of artifacts.

As for artifact rejection, epochs in which the variations in the EEG and EOG signals were larger than ± 80 μV were automatically excluded from the on-line averaging. The percentage of rejected epochs was 10.0% in the 8-year-olds, 20.6% in the 9-year-olds, 25.5% in the 10-year-olds, 25.7% in the 11-year-olds, 14.0% in the 12-year-olds, and 14.8% in the 13-year-olds.

More than 40 ERP trials were averaged for each condition. However, the number of averaged trials was less than 40 for all conditions in three 10-year-olds, one 11-year-old, and one 13-year-old.

As for the ERP analysis, the time window for the analysis ran from 100 ms before to 400 ms after stimulus onset in order to minimize the influence of artifacts, and the data obtained during the 100 ms before stimulus onset were used as the baseline. We analyzed the N170 component from 50 ms before its maximum (negative) to 50 ms after its maximum (negative) using the grand-average waveforms recorded for each group by the T5 (left) and T6 (right) electrodes. Peak latency was determined individually at the point after stimulus onset at which the N170 amplitude for each condition peaked. In the maximal N170 amplitude analysis, we used both the baseline-to-peak and peak-to-peak methods. As additional analyses, the latency and amplitude (baseline-to-peak) of the P100 component were measured at the O1 (left) and O2 (right) electrodes. N170 was longer in duration and/or had at least two peaks in many of the 8–11-year-old children, and the positive component that followed N170 could not always be clearly identified. Therefore, we did not measure the positive component that followed N170.

The data were analyzed by repeated-measures analysis of variance (ANOVA), and *stimulus condition* (upright face, inverted face, or eyes), *electrode* (P100: O1 or O2, N170: T5 or T6), and *age* (8-, 9-, 10-, 11-, 12-, or 13-years-old) were included as factors. Huynh and Feldt’s correction was used if the sphericity assumption was violated. The Bonferroni test was used for *post hoc* analyses, and *p*-values of < 0.05 were considered significant.

## Results

### P100

#### P100 Waveform

**Figure 2:**
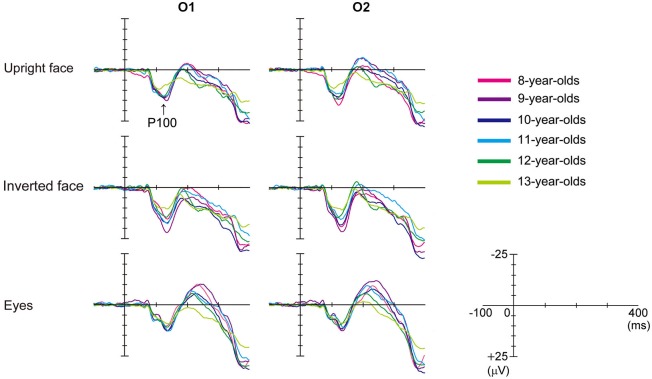
**Differences in the grand-averaged waveforms recorded by the O1 (left) and O2 (right) electrodes among the three stimulus conditions (upright face, inverted face, and eyes) in each age group (8–13-year-olds).** P100 was a positive deflection between 100–200 ms for all ages in this figure. P100 amplitude decreased as age increased.

Figure 2 shows the grand-averaged waveforms obtained for the 8–13-year-old children in all stimulus conditions (upright face, inverted face, and eyes) by the O1 (left) and O2 (right) electrodes. Table 1 shows the mean (and standard deviation) P100 latency and amplitude (baseline-to-peak) values obtained for each age group in each condition.

**Table 1:** **Latency and amplitude (baseline-to-peak) of P100 at O1 and O2 in the upright face, inverted face, and eyes stimulus conditions**.

	Upright face		Inverted face		Eyes	
	O1 (ms)	O2 (ms)	O1 (ms)	O2 (ms)	O1 (ms)	O2 (ms)
**Latency**
8-year-olds	128.6 ± 27.5	123.4 ± 12.4	141.5 ± 21.9	131.5 ± 18.1	138.5 ± 28.3	135.4 ± 26.2
9-year-olds	128.6 ± 14.9	120.0 ± 15.7	134.3 ± 12.6	134.6 ± 10.8	132.1 ± 19.5	128.9 ± 17.8
10-year-olds	120.1 ± 17.1	118.8 ± 14.6	132.2 ± 21.9	129.7 ± 14.3	136.5 ± 24.7	132.5 ± 20.2
11-year-olds	122.5 ± 16.1	120.3 ± 17.1	132.0 ± 14.0	122.4 ± 19.3	136.3 ± 12.3	126.6 ± 24.0
12-year-olds	120.7 ± 11.7	119.6 ± 11.6	128.0 ± 19.5	119.6 ± 14.6	136.7 ± 22.1	128.7 ± 21.0
13-year-olds	115.0 ± 16.8	116.2 ± 18.0	126.2 ± 18.9	125.9 ± 19.3	128.4 ± 22.9	124.8 ± 19.9
**Amplitude**	**(μV)**	**(μV)**	**(μV)**	**(μV)**	**(μV)**	**(μV)**
8-year-olds	18.3 ± 7.2	20.5 ± 5.4	18.6 ± 9.1	21.3 ± 8.9	15.7 ± 6.2	15.2 ± 7.2
9-year-olds	18.6 ± 8.3	17.8 ± 6.6	24.6 ± 7.1	23.8 ± 5.4	16.0 ± 8.1	15.6 ± 7.1
10-year-olds	18.2 ± 6.3	17.8 ± 5.2	21.0 ± 7.4	21.2 ± 7.8	15.8 ± 6.3	14.3 ± 5.6
11-year-olds	16.8 ± 9.2	15.6 ± 7.6	20.0 ± 7.5	17.8 ± 6.0	15.8 ± 7.6	14.8 ± 5.2
12-year-olds	15.2 ± 6.8	16.7 ± 7.8	16.0 ± 6.4	16.9 ± 8.4	13.2 ± 6.8	12.9 ± 8.2
13-year-olds	11.3 ± 4.2	12.4 ± 4.5	13.4 ± 4.1	14.3 ± 4.5	11.4 ± 5.1	11.8 ± 5.4

*Data are presented as the mean and standard deviation for each age group*.

#### P100 Latency

The *stimulus condition* (*F* = 20.20, *p* < 0.01, partial *η*^2^ = 0.210) and *electrode* (*F* = 15.92, *p* < 0.01, partial *η*^2^ = 0.173) had significant effects on the latency of P100. P100 latency was shortest after the presentation of the upright face stimuli, and shorter P100 latency values were detected at the O2 electrode (right hemisphere) than at the O1 electrode (left hemisphere). *Age* (*F* = 0.82, *p* > 0.05, partial *η*^2^ = 0.051) did not have a significant effect on P100 latency, nor did any of the interactions between the parameters.

We investigated the differences in P100 latency among the *stimulus conditions* in each age group using *post-hoc* analysis. P100 latency was significantly longer after the presentation of the inverted face stimuli than after the presentation of the upright face stimuli in the 9-, 10-, and 13-year-old children (9-year-olds: *p* < 0.01, 10-year-olds: *p* < 0.05, 13-year-olds: *p* < 0.05). In addition, P100 latency was significantly longer after the presentation of the eyes stimuli than after the presentation of the upright face stimuli in 10- and 13-year-old children (*p* < 0.05). The *stimulus condition* did not have a significant effect on P100 latency in the 8- or 11–12-year-old children (8-year-olds: *F* = 1.573, *p* > 0.05, partial *η*^2^ = 0.136; 11-year olds: *F* = 2.885, *p* > 0.05, partial *η*^2^ = 0.208; 12-year-olds: *F* = 2.511, *p* > 0.05, partial *η*^2^ = 0.218).

#### P100 Amplitude

The *stimulus condition* (*F* = 31.87, *p* < 0.01, partial *η*^2^ = 0.295) and *age* (*F* = 3.32, *p* < 0.01, partial *η*^2^ = 0.179) had significant effects on P100 amplitude. P100 amplitude was greatest after the presentation of the inverted face stimuli and decreased as age increased. However, the effect of *electrode* (*F* = 0.01, *p* > 0.05, partial *η*^2^ = 0.000) on the amplitude of P100 was not significant. The *stimulus condition* ×* electrode* (*F* = 3.18, *p* < 0.05, partial *η*^2^ = 0.040) and* stimulus condition* ×* age* (*F* = 2.03, *p* < 0.05, partial *η*^2^ = 0.118) interactions also had significant effects on P100 amplitude.

We investigated the differences in P100 amplitude among the *stimulus conditions* in each age group using *post-hoc* analysis. P100 amplitude was significantly greater after the presentation of the inverted face stimuli than after the presentation of the eyes stimuli in the 8–11-year-old children (8-year-olds: *p* < 0.05, 9-year-olds: *p* < 0.01, 10-year-olds: *p* < 0.01, 11-year-olds: *p* < 0.05). In addition, P100 amplitude was significantly greater after the presentation of the inverted face stimuli than after the presentation of the upright face stimuli in the 9-year-old children (*p* < 0.05). In 12-year-old children, the *stimulus condition* did not have a significant effect on P100 amplitude (*F* = 2.607, *p* > 0.05, partial *η*^2^ = 0.225). In 13-year-old children, the *stimulus condition* had a significant effect on P100 amplitude (*F* = 4.565, *p* < 0.05, partial *η*^2^ = 0.222). P100 tended to exhibit a greater amplitude after the presentation of the inverted face stimuli than after the presentation of the upright face stimuli, but this difference was not significant (*p* = 0.061).

### N170

#### N170 Waveform

**Figure 3:**
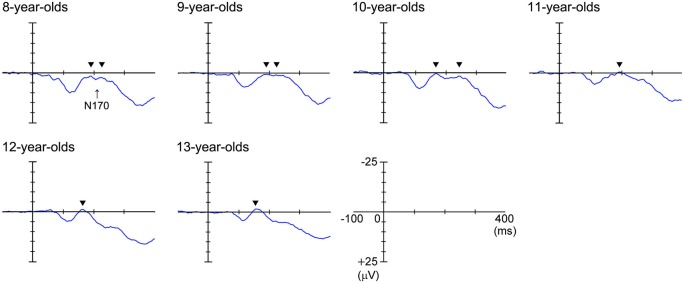
**Grand-averaged waveforms recorded at the T6 (right) electrode in response to the upright face stimuli.** N170 was a large negative deflection between 150–250 ms for all ages in this figure. The large negative deflection (N170) was longer in duration and/or had at least two peaks in the 8–11-year-old children, whereas it was sharp and had one peak in the 12–13-year-old children. The black arrows indicate the abovementioned peaks.

**Figure 4:**
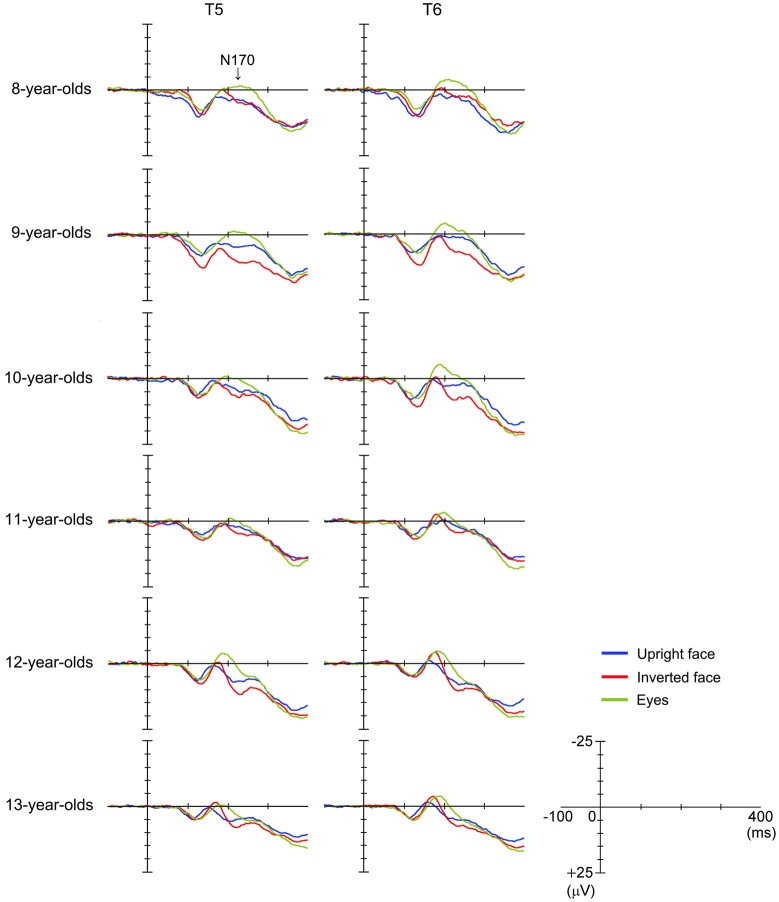
**Differences in grand-averaged waveforms recorded at the T5 (left) and T6 (right) electrodes among the three stimulus conditions (upright face, inverted face, and eyes) in each age group (8–13-year-olds)**.

Figure 3 shows the grand-averaged waveforms obtained with the upright face stimuli by the T6 (right) electrode in each age group. The large negative deflection (N170) observed after the presentation of the upright face stimuli was longer in duration and/or had at least two peaks in the 8–11-year-old children, whereas it was sharp and had a single peak in the 12–13-year-old children (Figure 3). Figure 4 shows the grand-averaged waveforms recorded for the 8- to 13-year-old children in each stimulus condition (upright face, inverted face, or eyes) by the T5 (left) and T6 (right) electrodes. The grand-averaged waveforms exhibited a large negative deflection in all age groups and all stimulus conditions. Table 2 shows the mean (and standard deviation) N170 latency and amplitude (baseline-to-peak and peak-to-peak) values obtained in each stimulus condition (upright face, inverted face, and eyes) by the T5 (left) and T6 (right) electrodes.

**Table 2:** **Latency and amplitude (baseline-to-peak and peak-to-peak values) of N170 at the T5 and T6 electrodes in the upright face, inverted face, and eyes stimulus conditions**.

	Upright face		Inverted face		Eyes	
	T5 (ms)	T6 (ms)	T5 (ms)	T6 (ms)	T5 (ms)	T6 (ms)
**Latency**
8-year-olds	217.2 ± 32.3	203.7 ± 27.8	200.4 ± 34.6	207.5 ± 24.2	222.5 ± 24.4	217.1 ± 24.2
9-year-olds	207.3 ± 40.0	204.3 ± 28.7	198.5 ± 32.3	200.3 ± 27.3	227.3 ± 28.8	210.3 ± 26.9
10-year-olds	179.4 ± 24.2	181.5 ± 26.2	185.3 ± 22.4	182.5 ± 17.4	207.1 ± 23.5	197.3 ± 19.0
11-year-olds	205.8 ± 26.7	196.4 ± 27.2	196.3 ± 29.4	189.0 ± 23.9	209.3 ± 22.7	207.5 ± 23.6
12-year-olds	170.0 ± 21.7	172.2 ± 20.3	173.8 ± 8.8	175.2 ± 11.1	195.0 ± 15.0	188.0 ± 16.1
13-year-olds	160.7 ± 18.0	163.2 ± 16.1	169.4 ± 11.1	173.7 ± 13.9	177.1 ± 22.6	186.4 ± 15.5
**Amplitude (baseline-to-peak)**	(**μV**)	(**μV**)	(**μV**)	(**μV**)	(**μV**)	(**μV**)
8-year-olds	0.2 ± 4.7	−2.4 ± 4.1	−1.0 ± 5.5	−4.2 ± 2.9	−4.5 ± 5.3	−8.1 ± 4.5
9-year-olds	−1.0 ± 7.9	−3.3 ± 8.5	2.8 ± 8.5	−1.9 ± 8.9	−4.7 ± 5.9	−6.7 ± 6.9
10-year-olds	−1.4 ± 3.9	−1.7 ± 4.1	−0.8 ± 5.4	−2.1 ± 4.6	−3.7 ± 4.9	−6.8 ± 5.0
11-year-olds	−2.1 ± 3.7	−3.3 ± 7.2	−1.0 ± 4.3	−5.4 ± 5.0	−3.2 ± 4.6	−5.7 ± 8.6
12-year-olds	−1.0 ± 5.3	−3.1 ± 5.5	−1.8 ± 7.2	−6.0 ± 5.9	−6.6 ± 7.6	−6.6 ± 5.4
13-year-olds	−1.6 ± 3.8	−3.9 ± 5.3	−3.2 ± 4.1	−5.5 ± 4.6	−3.3 ± 4.3	−5.9 ± 6.4
**Amplitude (peak-to-peak)**	(**μV**)	(**μV**)	(**μV**)	(**μV**)	(**μV**)	(**μV**)
8-year-olds	12.7 ± 5.9	14.3 ± 4.2	11.9 ± 8.1	17.1 ± 3.1	13.8 ± 6.2	17.4 ± 5.2
9-year-olds	11.8 ± 5.7	14.1 ± 7.0	11.9 ± 5.5	15.9 ± 6.4	14.3 ± 5.9	16.2 ± 7.8
10-year-olds	9.7 ± 3.9	11.3 ± 4.9	9.9 ± 4.9	14.7 ± 7.6	11.9 ± 5.5	16.4 ± 8.1
11-year-olds	8.7 ± 4.8	10.6 ± 6.4	9.8 ± 5.6	14.4 ± 6.5	11.2 ± 5.4	13.1 ± 8.3
12-year-olds	9.2 ± 5.6	11.4 ± 4.7	12.0 ± 6.7	15.0 ± 6.7	14.4 ± 5.2	13.4 ± 6.9
13-year-olds	7.9 ± 4.3	10.5 ± 6.2	10.7 ± 5.5	12.8 ± 7.2	10.3 ± 4.6	13.2 ± 6.7

*Data are shown as the mean and standard deviation for each age group*.

#### N170 Latency

The *stimulus condition* (*F* = 33.24, *p* < 0.01, partial *η*^2^ = 0.304) and* age* (*F* = 11.44, *p* < 0.01, partial *η*^2^ = 0.429) had significant effects on N170 latency. N170 latency was greatest after the presentation of the eyes stimuli, and decreased as age increased. The effect of *electrode* (*F* = 1.98, *p* > 0.05, partial *η*^2^ = 0.025) on N170 latency was not significant, nor were any of the interactions between the parameters.

We investigated the differences in N170 latency among the *stimulus conditions* in each age group using *post-hoc* analysis. *Stimulus condition* (*F* = 1.93, *p* > 0.05, partial *η*^2^ = 0.161) did not have a significant effect on N170 latency in the 8-year-old children. The N170 latency observed after the presentation of the eyes stimuli was significantly longer than those observed after the presentation of the upright or inverted face stimuli in the 10- and 12-year-old children (*p* < 0.01). On the other hand, the N170 latency observed after the presentation of the eyes stimuli was significantly longer than that observed after the presentation of the inverted face stimuli in the 9- and 11-year-old children (9-year-olds: *p* < 0.05, 11-year-olds: *p* < 0.01). Significant differences in N170 latency were observed among all three stimulus conditions in the 13-year-old children, with the upright face stimuli producing the shortest latency and the eyes stimuli producing the longest latency (*p* < 0.01).

#### N170 Amplitude According to the Baseline-to-Peak Method

The *stimulus condition* (*F* = 33.04, *p* < 0.01, partial *η*^2^ = 0.303) and *electrode* (*F* = 22.57, *p* < 0.01, partial *η*^2^ = 0.229) had significant effects on N170 amplitude, while the effect of *age* (*F* = 0.29, *p* > 0.05, partial *η*^2^ = 0.018) was not significant. The *stimulus condition* × *age* interaction (*F* = 2.79, *p* < 0.01, partial *η*^2^ = 0.155) also had a significant effect on N170 amplitude. N170 amplitude was greatest after the presentation of the eyes stimuli, and larger N170 amplitude values were detected at the T6 electrode (right hemisphere) than at the T5 electrode (left hemisphere).

We investigated the differences in N170 amplitude according to the baseline-to-peak method among the *stimulus conditions* in each age group using *post-hoc* analysis. A significant effect of *stimulus condition* was detected in the 8–10- and 12-year-old children. In addition, N170 exhibited a significantly greater amplitude after the presentation of the eyes stimuli than after the presentation of the upright face or inverted face stimuli in the 8–10- and 12-year-old children (upright face: 8-year-olds: *p* < 0.01, 9-year-olds: *p* < 0.05, 10-year-olds: *p* < 0.01, 12-year-olds: *p* < 0.05; inverted face: 8-year-olds: *p* < 0.05, 9-year-olds: *p* < 0.01, 10-year-olds: *p* < 0.05, 12-year-olds: *p* < 0.01). In the 13-year-old children, N170 tended to exhibit a greater amplitude after the presentation of the eyes stimuli than after the presentation of the upright face stimuli, which was similar to the results we obtained for the 12-year-old children.

#### N170 Amplitude According to the Peak-to-Peak Method

The *stimulus condition* (*F* = 31.20, *p* < 0.01, partial *η*^2^ = 0.291) and *electrode* (*F* = 13.49, *p* < 0.01, partial *η*^2^ = 0.151) had significant effects on N170 amplitude, while the effect of *age* (*F* = 1.46, *p* > 0.05, partial *η*^2^ = 0.088) was not significant. The *stimulus condition* × *electrode* interaction (*F* = 6.33, *p* < 0.01, partial *η*^2^ = 0.077) also had a significant effect on N170 amplitude. N170 amplitude was smallest after the presentation of the upright face stimuli, and greater N170 amplitude values were detected at the T6 electrode (right hemisphere) than at the T5 electrode (left hemisphere), as was found using the baseline-to-peak method.

We investigated the differences in N170 amplitude according to the peak-to-peak method among the *stimulus conditions* in each age group using *post-hoc* analysis. A significant effect of *stimulus condition* was detected in the 10–13-year-old children. The amplitude of N170 was significantly greater after the presentation of the inverted face or eyes stimuli than after the presentation of the upright face stimuli in the 10-, 12-, and 13-year-old children (inverted face: 10-year-olds: *p* < 0.05, 12-year-olds: *p* < 0.01, 13-year-olds: *p* < 0.05; eyes: 10-year-olds: *p* < 0.01, 12-year-olds: *p* < 0.05, 13-year-olds: *p* < 0.01). The amplitude of N170 was significantly greater after the presentation of the inverted face stimuli than after the presentation of the upright face stimuli in the 11-year-old children (*p* < 0.05).

#### Latency Differences Between N170 and P100

We assessed the latency differences between N170 and P100 in the left (N170 at T5 and P100 at O1) and right (N170 at T6 and P100 at O2) hemispheres. Table 3 shows the mean (and standard deviation) latency differences between the N170 and P100 components in the left and right hemispheres.

**Table 3:** **Latency differences between N170 and P100 in the left (N170 at T5 and P100 at O1) and right (N170 at T6 and P100 at O2) hemispheres in the upright face, inverted face, and eyes stimulus conditions**.

	Upright face		Inverted face		Eyes	
	Left (ms)	Right (ms)	Left (ms)	Right (ms)	Left (ms)	Right (ms)
8-year-olds	88.6 ± 44.3	80.3 ± 33.0	58.9 ± 41.3	76.0 ± 28.1	84.0 ± 37.9	81.8 ± 31.8
9-year-olds	78.7 ± 42.4	84.3 ± 38.0	64.2 ± 31.0	65.7 ± 26.6	95.2 ± 28.7	81.4 ± 29.7
10-year-olds	59.3 ± 23.1	62.7 ± 24.8	53.1 ± 16.8	52.8 ± 20.8	70.7 ± 30.5	64.7 ± 24.4
11-year-olds	83.5 ± 25.2	76.1 ± 26.6	64.3 ± 36.4	66.6 ± 21.4	73.0 ± 28.2	80.9 ± 27.1
12-year-olds	49.3 ± 19.4	52.6 ± 22.7	45.8 ± 18.1	55.6 ± 16.1	58.4 ± 18.7	59.3 ± 18.4
13-year-olds	45.7 ± 16.7	47.0 ± 12.4	43.1 ± 15.5	47.8 ± 17.7	48.8 ± 22.4	61.6 ± 17.0

*Data are shown as the mean and standard deviation for each age group*.

The resultant data were analyzed by repeated-measures ANOVA with *stimulus condition* (upright face, inverted face, or eyes), *hemisphere* (left or right), and *age* (8-, 9-, 10-, 11-, 12-, or 13-years-old) as factors. The latency difference between N170 and P100 was significantly affected by the *stimulus condition* (*F* = 11.11, *p* < 0.01, partial *η*^2^ = 0.128) and *age* (*F* = 7.22, *p* < 0.01, partial *η*^2^ = 0.322). The effect of* hemisphere* (*F* = 0.80, *p* > 0.05, partial *η*^2^ = 0.010) was not significant. In addition, the *stimulus condition* × *hemisphere* × *age* interaction (*F* = 1.925, *p* < 0.05, partial *η*^2^ = 0.112) had a significant effect on the latency differences between N170 and P100. None of the other interactions had similar effects. The latency differences between N170 and P100 were smallest after the presentation of the inverted face stimuli and decreased as age increased.

## Discussion

The results of the present study can be summarized as follows: (1) P100 amplitude decreased significantly as age increased; (2) N170 latency significantly decreased as age increased, and the latency differences between N170 and P100 significantly decreased as age increased; (3) N170 exhibited a significantly longer latency after the presentation of the eyes stimuli than after the presentation of the upright face stimuli in the 10- and 12-year-old children; (4) Significant differences in N170 latency were observed among all three stimulus types in the 13-year-old children; (5) N170 exhibited a significantly greater amplitude after the presentation of the eyes stimuli than after the presentation of the upright face stimuli in the 8–10- and 12-year-old children.

The reduction in P100 amplitude observed with age in the present study agreed with the results of our previous study (Miki et al., 2011). Based on the findings of previous studies (Pastò and Burack, 1997; Skoczenski and Norcia, 2002; Want et al., 2003; Betts et al., 2006; Crookes and McKone, 2009), we speculate that age-related changes in P100 reflect the general development of sensory and/or cognitive function.

The reduction in N170 latency observed with age in the present study was consistent with the findings of a previous study (Taylor et al., 2004), and the latency differences between N170 and P100 significantly decreased as age increased. Based on the findings of previous studies, we speculate that: (1) the reduction in N170 latency seen with age was not influenced by P100 latency; and (2) the observed reduction in N170 latency was driven by perceptual and cognitive development (Mitchell and Neville, 2004; Doucet et al., 2005), which differs from the underlying mechanism that is considered to be responsible for changes in P100 latency. Previous fMRI studies have observed developmental changes in face-specific areas of the brain (the FFA, OFA, and STS) (Scherf et al., 2007) and the extended face-processing network (e.g., the inferior frontal gyrus) (Joseph et al., 2011). Therefore, we speculate that changes in N170 latency might reflect developmental changes in areas of the brain related to face perception.

In the present study, we found that after the presentation of the upright face stimuli the N170 component was longer in duration and/or had at least two peaks in the 8–11-year-old children, whereas it was sharp and had one peak in the 12–13-year-old children. In a previous study (Taylor et al., 2004), N170 was composed of two subcomponents in about two thirds of young children (less than 10–11-years-old). The first N170 component (N170a) was only present in some young children and was rarely detected in older children. In addition, it had a flatter developmental curve and reached adults levels at a younger age. In contrast, the second N170 component (N170b) showed a prolonged and steeper maturation curve, the latency of which was markedly longer in the younger age groups. Taylor et al. (2004) suggested that these two subcomponents might reflect different functional sources in the temporo-occipital and lateral temporal cortices. The N170 components observed in the 8- to 11-year-old children in the present study were consistent with those described in the above study.

When we used the peak-to-peak method to analyze our data, we found that the amplitude of N170 was affected by the previous component; i.e., P100. In this study, the *stimulus condition* and *electrode* had significant effects on N170 amplitude according to both the peak-to-peak and baseline-to-peak methods, while the effect of *age* was not significant. On the other hand, the *stimulus condition* × *age* interaction was demonstrated to have a significant effect on N170 by the baseline-to-peak method, but not the peak-to-peak method. Therefore, we consider that the baseline-to-peak method might be more valuable for investigating developmental changes in N170 amplitude than the peak-to-peak method. In addition, we consider that it is important to analyze both P100 and N170 and to perform comparisons between N170 amplitude data obtained with the baseline-to-peak and peak-to-peak methods during studies of the developmental changes in face perception-related N170 signals. The amplitude of N170 might be affected by the amplitude of P100, and our findings regarding the relationship between P100 and N170 suggest that the development of face perception (reflected by N170) is based on the development of more basic visual functions (indicated by P100).

Previous face inversion studies have shown that inversion disrupted holistic and/or configural processing during face perception, but had little or no effect when the presented stimuli were processed featurally (Freire et al., 2000; Maurer et al., 2002). Mondloch et al. (2002) speculated that configural processing might only approach adult levels after children reach 10 years of age and might develop very slowly. In the present study, the inversion effect was only observed in the 13-year-old children. Based on the abovementioned studies, we speculate that: (1) the upright face stimuli were processed holistically and/or configurally in the 13-year-old children, but not in those younger than 12-years-old; and (2) that at 13 years of age the pattern of responses to facial stimuli becomes similar to those described in adult studies (Watanabe et al., 2003).

The results of the present study were generally consistent with the findings of previous studies (Taylor et al., 1999, 2004; Itier and Taylor, 2004a,b). However, our findings differed from those of previous studies with regard to the age at which the adult response pattern was observed. We consider that cultural differences might have been one of the reasons for this. Many studies have examined cultural differences in face perception processing. Blais et al. (2008) monitored the eye movements of Western Caucasians and East Asians during learning and recognition in a face recognition task and a face categorization by race task, and suggested that the Western Caucasians consistently fixated on the eye region and partially concentrated on the mouth, whereas the East Asian subjects fixated more on the central region of the face. In addition, an fMRI study demonstrated higher facial selectivity in Western individuals in the left FFA and a greater degree of right-sided lateralization in the FFA in East Asians. These findings were consistent with an analytical style of face processing in Western individuals and a holistic processing style in East Asians (Goh et al., 2010). Based on the above studies, we speculated that our findings might differ from those of previous developmental studies of N170 due to variations in face perception processing between Japanese and Western children. We consider that our division of the subjects into one-year age groups, rather than the two-year age groups used in previous studies, might also partially explain these differences.

In the present study, we investigated the development of face perception in Japanese children, and the results obtained led to speculation regarding the changes in face processing strategies (holistic and/or configural vs. feature-based) that occur during childhood and cultural differences in face perception strategies. This study had several limitations, with the most important being that we did not examine children under 7-years-old or over 14-years-old. However, we detected a marked change in the development of face perception during childhood in this study. The second limitation was associated with the presented stimuli. The use of target images including facial features might have resulted in some bias, for example, a bias in the way the children visually explored the facial stimuli (it might have encouraged them to focus on the eyes or the nose/mouth regions), which could have affected the processing strategies they used to perceive faces. In addition, some of the images of kettles with mustaches, glasses, and fake noses that were presented as target stimuli might have been perceived as face stimuli because they were only presented for a short period (250 ms). However, our results were consistent with those of previous studies involving children and adults. Therefore, I consider that the target stimuli used in this study ensured that the experimental task was an implicit face perception task, although they might have introduced some element of bias. The third limitation was that we only examined Japanese children. As described above, cultural differences in the development of face perception might be important, and we intend to investigate this issue in more detail in future studies. Another limitation was that the properties of the presented face (upright and inverted) and eyes stimuli, e.g., their luminance, differed. P100 might be affected by the properties of visual stimuli rather than differences in the type of processing involved in face perception and so might have varied among the stimulus conditions in the present study. In future studies, we intend to use stimuli with similar properties and investigate the relationships between P100 and N170 during face processing.

## Author Contribution

SW designed the experiments. YH and SW performed the experiments and EEG data recording. YH performed the EEG data analysis and statistical analyses. YT made the program used for the stimulus presentation in this study. KM performed the statistical analyses and wrote the manuscript. RK made suggestions about this study and had overall responsibility for it.

## Conflict of Interest Statement

The authors declare that the research was conducted in the absence of any commercial or financial relationships that could be construed as a potential conflict of interest.
